# New Technologies and Strategies for Grapevine Breeding Through Genetic Transformation

**DOI:** 10.3389/fpls.2021.767522

**Published:** 2021-11-25

**Authors:** Gabriela Campos, Constanza Chialva, Silvana Miras, Diego Lijavetzky

**Affiliations:** Instituto de Biología Agrícola de Mendoza (IBAM, CONICET-UNCuyo), Almirante Brown 500, M5528AHB. Chacras de Coria, Mendoza, Argentina

**Keywords:** grapevine, genetic transformation, nanotechnology, regeneration, transcription factors, development regulators, *Vitis vinifera*, gene editing

## Abstract

Grapevine, as other woody perennials, has been considered a recalcitrant crop to produce transgenic plants. Since the production of transgenic and/or edited plants requires the ability to regenerate plants from transformed tissues, this step is often the biggest bottleneck in the process. The objective of this work is to review the state of the art technologies and strategies for the improvement of grapevine transformation and regeneration, focusing on three aspects: (i) problems associated with grapevine transformation; (ii) genes that promote grapevine regeneration; and (iii) vehicles for gene delivery. Concerning the first aspect, it is well documented that one of the main factors explaining the low success rate in obtaining transgenic plants is the regeneration process. After transgenic integration into receptor cells, tissue culture is required to regenerate transgenic seedlings from transformed cells. This process is time consuming and often requires the addition of environmentally damaging reagents (antibiotics and herbicides) to the culture medium to select transgenic plants. On the other hand, the expression of genes such as the so-called developmental regulators (DR), which induce specific development programs, can be used to avoid traditional tissue culture methods. The ectopic expression of specific combinations of DR in somatic cells has the potential to induce *de novo* meristems in diverse crops, including grapevine. Successful genome editing by *de novo* reprogramming of plant meristems in somatic tissues has been reported. Moreover, it has been shown that the expression of certain transcription factors can increase the regeneration efficiency in wheat, citrus, and rice. Finally, recent reports showed the use of nanoparticles, such as carbon dots (CDs), as an attractive alternative to *Agrobacterium*- and biolistic-mediated plant genetic transformation. In this way, the use of antibiotics in culture media is avoided, overcoming the loss of viability of plant tissues and accelerating the regeneration processes. It has been shown that CDs can act as a vehicle to transport plasmids to plant cells in transient transformation in several crops without negative impacts on photosynthesis or growth. Based on these advances, it is possible to combine these new available strategies and technologies to overcome the regeneration problems of species such as grapevine and other crops considered as recalcitrant.

## Introduction

Grapevine is one of the most widespread fruit crops in the world, with a production of about 77.1 million tons (FAOSTAT, 2019). It is cultivated both for the manufacture of wine and for its consumption as fresh fruit, and to a lesser extent to produce raisins, juices, and spirit drinks, the first use being the one for which it has more hectares allocated. Actual and future environmental conditions impose the decisions and choices regarding the management of the crop, following a path toward more sustainable alternatives. The problems related to climate change and the spread of numerous diseases require addressing solutions that can be found in the natural genetic variation of the genus *Vitis* (Vezzulli et al., 2019). Although grapevine can be improved through conventional breeding, it is a difficult and time-consuming process, due to the 2–3 years generation cycle and the long period of time required for the selection and testing of reliable progeny. Also, for grapevine, whose varieties are highly heterozygous, this way is even more difficult (Gray and Meredith, 1992).

Several years ago, genetic engineering emerged as an outstanding tool for the improvement of plants (Anderson et al., 2019). Genetic transformation offers the possibility of genetically modifying plants to improve agronomic traits of interest without altering the varietal identity using recombinant DNA technology, such as the transfer of resistance to diseases or herbicides to established crops. Grapevine was considered recalcitrant to genetic transformation, since one of its problems is the regeneration of plants from the tissues used for genetic transformation (Mullins et al., 1990; Nakano et al., 1994). The regeneration rate of grapevine plants after transformation and selection by antibiotics ranges between 10 and 30% of the total transformed material, and the transformation efficiency, which varies according to the genotype, down to 1%, although a 33% has been described (Torregrosa et al., 2015).

Accordingly, overcoming the problems related to tissue regeneration is one of the most essential challenges in the generation of transgenic and edited grapevine plants. In this sense, several factors have been identified that should be reviewed: the grapevine genotype; the type of tissue used to obtain the explants; the transformation methodology; the availability of regenerative transformable material, and the selection process/procedure based on antibiotics. Grapevine genetic transformation was mainly performed by infection with *Agrobacterium tumefaciens* (Torregrosa et al., 2015), and to a lesser extent through the biolistic techniques (Kikkert et al., 2001; Vidal et al., 2010).

The first critical factor in grapevine transformation is the production of highly regenerative transformable material, where the regeneration efficiency greatly depends on the different genotypes (Gray and Meredith, 1992). Somatic embryogenesis is the preferred regeneration procedure for the genetic transformation of grapevine. The source of starting material, the type, and the quality of the embryogenic cultures are key factors for a successful transformation (Martinelli and Gribaudo, 2001). However, despite these limitations, it has been possible to produce grapevine varieties resistant to fungal, viral, and bacterial diseases (Mauro et al., 1995; Scorza et al., 1996; Agüero et al., 2006; Nirala et al., 2010; Capriotti et al., 2020).

Beyond grapevine, the production of transgenic and edited plants requires, for most crops, the ability to regenerate plants from transformed tissues. This step is another critical factor, often reported as the biggest bottleneck in the process (Altpeter et al., 2016). After transgenic integration into recipient cells, tissue culture protocols are required to regenerate transgenic seedlings from transformed cells. This process is time consuming, usually several months, and generally requires the addition of expensive and environmentally harmful reagents (antibiotics and herbicides) to the growing medium to select the putative transgenic plants. The overexpression of certain transcription factors has been recently reported (Debernardi et al., 2020; Maher et al., 2020) as an interesting alternative to improve the transformation and regeneration processes of plants (including grapevine).

In recent years, new breeding technologies (NBT), such as gene editing *via* CRISPR-Cas9, have emerged as innovative genetic improvement tools for various crops of agronomic importance (Dalla Costa et al., 2017, 2019). The key elements in this system are Cas nucleases and CRISPR RNAs. The Cas9 endonuclease can cut at specific DNA target sites with the help of two small RNA molecules called CRISPR RNA (crRNA) and “*trans*-encoded” CRISPR RNA (tracrRNA). These two molecules can be fused artificially to form a chimeric RNA molecule called “single guide RNA” (sgRNA) (Mali et al., 2013; Qi et al., 2013). In conjunction with Cas9, sgRNA can form an “RNA-guided endonuclease,” a high precision tool capable of strategically introducing targeted mutations in the host genome (Jinek et al., 2012; Samanta et al., 2016).

On the other hand, alternative technologies are also emerging for the delivery of engineered genes into the plant cells. Carbon dots (CDs) were described as almost spherical water-soluble nanoparticles (NP) consisting of crystalline carbon domains synthesized from cheap starting materials such as peptides, carbohydrates, and, in general, a wide range of carbon sources (Li et al., 2012; Zheng et al., 2015; Peng et al., 2017). They have multiple advantages, such as being easy to obtain and for displaying an efficient plant cell uptake. It has been shown that CDs can act as a vehicle to transport plasmids to plant cells in transient transformation in several important crop species without negative impacts on photosynthesis or growth (Doyle et al., 2019; Schwartz et al., 2020).

Along with a thorough report of commonly known limitations related to grapevine transformation and regeneration, we primarily present in this review a detailed description of new alternative technologies that could provide solutions to overcome these drawbacks.

## Main Limitations Affecting Traditional Transformation and Regeneration in Grapevine

Since the genotype is one of the most influential factors in the success of a transformation protocol, the effects of the genetic background on the efficiency of plant regeneration and the corresponding culture conditions have been extensively studied (Dalla Costa et al., 2019). Thus, it has been reported that each genotype shows specific sensitivity to the infection with *Agrobacterium*, as well as to the antibiotics used to eliminate the bacteria, and/or to those used to select transgenic events (Zhou et al., 2014). The grapevine genetic background also influences somatic embryogenesis, the regeneration method most used in genetic engineering protocols in this crop. Several procedures have been developed for somatic embryogenesis from grapevine genotypes, and much research has been carried out using several plant tissues/organs as starting explants. The list of the plant parts widely used as suitable material for obtaining somatic embryos includes: ovaries (Yamamoto et al., 2000; Vidal et al., 2003, 2006; Gambino et al., 2005; Kikkert et al., 2005; López-Pérez et al., 2005); anthers (Franks et al., 1998; Gambino et al., 2005; Agüero et al., 2006; Prado et al., 2010); leaves (Martinelli et al., 1993; Nakano et al., 1994; Das et al., 2002); and, less frequently, stigmas and styles (Morgana et al., 2004; Carimi et al., 2005); stamen filaments (Nakajima and Matsuta, 2003; Acanda et al., 2013); nodal sections (Maillot et al., 2006, 2016); whole flowers (Gambino et al., 2007); mature seeds (Peiró et al., 2015); and tissues derived from vegetative structures, such as leaves and petioles (Martinelli et al., 1993; Das et al., 2002). The fact that most of the frequently used protocols are carried out using floral organs is indeed a strong limitation for obtaining somatic embryos, since the experiments can just be started at the flowering time. During the regeneration process, the germination of aberrant embryos and early germination can occur, which can also limit the obtaining of transformed grapevine plants since these embryos do not develop into normal plants. Among the most common aberrations, it is possible to find embryos without cotyledons, with different numbers of cotyledons, or with fused cotyledons, and trumpet-shaped or cauliflower-like cotyledons (Goebel-Tourand et al., 1993; Bornhoff et al., 2005; Li et al., 2006; Bharathy and Agrawal, 2008; Martinelli and Gribaudo, 2009; Peiró et al., 2015).

It has been proven that the synthesis and accumulation of reserve proteins during zygotic embryogenesis is regulated by abscisic acid and/or water stress (Dodeman et al., 1997). Therefore, to achieve a correct maturation of somatic embryos, two methodologies have been used: the exogenous application of abscisic acid (ABA) (Morris et al., 1990; Goebel-Tourand et al., 1993; Sholi et al., 2009) and the use of culture media with reduced water potential (Klimaszewska et al., 2000; Walker and Parrott, 2001; Kong et al., 2009; Buendía-González et al., 2012). Another strategy used has been the modulation of polyamine metabolism (Faure et al., 1991). The use of semipermeable cellulose acetate membranes has emerged as an effective alternative to improve the maturation of somatic embryos, and this is due to its ability to limit the availability of water for the embryo. In grapevines, it has been shown that the water stress produced by the membrane triggered an increase in endogenous levels of ABA, a fact that improved the maturation of somatic embryos (Acanda, 2015).

The choice of the *Agrobacterium* strain shows a significant effect on a successful transformation procedure of plant tissues. This factor also includes the corresponding bacterial culture conditions like density at the time of infection, coculture times, and the culture media used (Le Gall et al., 1994; Franks et al., 1998). One of the first *Agrobacterium* strains used for grapevine transformation was LBA4404 (Bouquet et al., 2008), showing low transformation efficiencies. To improve those transformation rates, hypervirulent *Agrobacterium* strains were later developed. Among them, EHA105 is currently the strain most used in grapevine transformation (Scorza et al., 1996; Franks et al., 1998; Iocco et al., 2001; Wang et al., 2005; Dutt et al., 2008; Dhekney et al., 2011; Dabauza et al., 2014; Li et al., 2015). AGL1, a hypervirulent *Agrobacterium* strain (Lazo et al., 1991) has also been used, verifying that agroinfiltration with this strain transformed all the tested genotypes (Urso et al., 2013).

Grapevine tissue necrosis is a regular problem that occurs during or after *Agrobacterium* transformation. This browning is triggered as a response to the bacteria and it was reported to be cultivar-specific (Bouquet et al., 2008). Polyphenols can be oxidized by air, peroxidases, or polyphenoloxidases. Peroxidases and polyphenoloxidases have been associated with mechanical injury and response to environmental stress. The stress response may involve the release of polyphenols from vacuoles and the *de novo* synthesis of phenol (Perl et al., 1996). These authors assume that the hypersensitive response could be due to the oxidation caused by high levels of peroxidase activity. Perl et al. (1996) improved plant viability and inhibited tissue necrosis in cv. “Superior Seedless” by using a combination of polyvinylpolypyrrolidone and dithiothreitol. A still unexplored alternative that could reduce this browning is the use of *Agrobacterium vitis* strains (*A. tumefaciens* biovar 3), which is a natural grapevine pathogen (Kikkert et al., 2001).

The differentiation of the positive transformation events among all the non-transformed plant tissues after infection with the *Agrobacterium* strain is a crucial step of the procedure. This is possible due to the action of marker genes, which are usually integrated together with the genes engineered to be expressed in the plant. There are two main types of marker genes: selection marker genes and reporter genes.

The use of marker genes allows the cells or plants carrying them to be selected in the presence of a selective agent, such as an herbicide or an antibiotic. The neomycin phosphotransferase (*nptII*) gene has been the most widely used in grapevine. This gene confers resistance to aminoglycoside antibiotics, such as kanamycin (Kan), paramomycin, or neomycin (Nakano et al., 1994; Scorza et al., 1995, 1996; Yamamoto et al., 2000; Iocco et al., 2001; Vidal et al., 2003, 2006; Bornhoff et al., 2005; Gambino et al., 2005; Wang et al., 2005; Agüero et al., 2006; Li et al., 2006, 2015; Dhekney et al., 2008, 2011; López-Pérez et al., 2008; Jin et al., 2009; Gago et al., 2011; Dabauza et al., 2014). Although kanamycin is the most widely selective agent used in grapevine transformation, this crop shows a high sensitivity to this antibiotic, and it is generally difficult to find a balance between the appropriate concentration for selection, without losing the viability of the embryos and plants. Another gene frequently used in grapevine transformation is hygromycin phosphotransferase (*hptI*), which confers resistance to the antibiotic hygromycin (Hyg) (Franks et al., 1998; Torregrosa et al., 2000; Fan et al., 2008; Nirala et al., 2010; Nookaraju and Agrawal, 2012; Dai et al., 2015). Different strategies have been assayed regarding the application timing and the optimal concentration of kanamycin (Franks et al., 1998; Iocco et al., 2001; Wang et al., 2005) and hygromycin (Franks et al., 1998; Fan et al., 2008; Nirala et al., 2010; Dai et al., 2015). Moreover, Saporta et al. (2014) made a comparison of Kan and Hyg in “Albariño” cell suspensions. Since grapevine antibiotic sensitivity showed a high genotype-specific behavior (Gray and Meredith, 1992; Torregrosa et al., 2000), sensitivity-specific assays are required for every new genetic transformation platform.

On the other hand, reporter genes give transformed plants an easily recognizable and measurable selection characteristic or phenotype. Once these genes are integrated, they allow us to know where they are expressed, in what quantity, when, and in which tissues they are transcribed. The most widely used reporter gene is the *uidA* gene that encodes β-glucuronidase (GUS) (Jefferson et al., 1987). This protein hydrolyzes substrates such as X-Gluc (5-bromo-4-chloro-3-indoxyl-beta-D-glucuronide) and causes a blue precipitate. The disadvantage of this method is that it is usually destructive. Alternatively, the green fluorescent protein gene (*GFP*), which encodes a protein that generates a chromophore emitting green fluorescence when excited by blue light or ultraviolet light, is commonly utilized as a non-destructive reporter system (Chiu et al., 1996). Both genes have been extensively used in grapevine genetic transformation (Baribault et al., 1989; Nakano et al., 1994; Scorza et al., 1995; Franks et al., 1998; Iocco et al., 2001; Das et al., 2002; Wang et al., 2005; Dutt et al., 2007, 2008; López-Pérez et al., 2008; Gago et al., 2011). More recently, He et al. (2020) reported *RUBY* as a non-invasive reporter that could be especially useful for monitoring gene expression in tissue culture experiments under sterile conditions in large crop plants such as fruit trees.

Alternatively to the plant transformation mediated by *A. tumefaciens*, several works reported the use of biolistic methodologies (Torregrosa et al., 2002; Vidal et al., 2003, 2006; Joubert et al., 2013). Biolistic is a physical transformation method, which consists of the high-speed projection of microparticles, usually gold and tungsten, impregnated with DNA (Sanford et al., 1987). The first grapevine work using biolistics was reported by Hébert et al. (1993), in which they transformed “Chancellor,” a *Vitis* complex interspecific hybrid. On the other hand, Scorza et al. (1995) carried out the first transformation of seedless table grapes using this technique. Nowadays, this technique is a useful transient expression tool for functional analysis in various plant materials, such as, cell suspension culture, leaf sections, and somatic embryos (Vidal et al., 2010; Jelly et al., 2014; Dalla Costa et al., 2019). A biolistic protocol based on the transient genetic transformation of “Cabernet Sauvignon” cell suspensions was developed by Torregrosa et al. (2002) to analyze the effect of anaerobiosis on the regulation of the expression of the *VvAdh* gene in response to anaerobiosis. Finally, biolistics transformation was also used to study the regulation of the defense gene *VvPGIP* in leaves sections of “Chardonnay” and ‘Thompson Seedless” somatic embryos (Joubert et al., 2013). One of the most important advantage of biolistic techniques with respect to the infection with *A. tumefaciens* is probably to skip the treatment with antibiotics to eliminate the bacteria, and in the case of grapevine, to avoid the before mentioned hypersensitivity reaction caused by infection. As disadvantages, low penetration depth, random integration, and putative damage to target tissue were also reported (Cunningham et al., 2018). Particle bombardment is still quite difficult to perform and requires the fine tuning of a series of critical variables such as helium pressure, particle diameter, cartridge preparation, or distance from target plant material. Additionally, purchasing a biolistic device and consumables can be expensive (Jelly et al., 2014).

## Status of the Grapevine Genetic Transformation Research

The technical and biological problems mentioned above, together with the strong rejection of the consumers and the regulation of the appellations of origin, have prevented the wide development of grapevine genetic transformation. Furthermore, due to the scarce natural genetic resistance/tolerance of *V. vinifera* genotypes, stable transformation has been mainly oriented to the improvement of resistance to pathogens and insects (Vivier and Pretorius, 2002; Table 1). Most of the reports on fungal resistance have focused on the use of pathogenesis related proteins (PR), among which, glucanases and chitinases stand out. On the other hand, the accumulation of phytoalexins and stilbenes has been a proven strategy used to obtain resistance to fungi (Fan et al., 2008; Dabauza et al., 2014; Dai et al., 2015). The improvement of plant resistance to bacteria has been targeted by using antimicrobial genes like lytic peptides (Scorza et al., 1996; Vidal et al., 2003, 2006; Dandekar et al., 2012; Li et al., 2015). The insertion of virus capsid proteins (virus coat proteins; CP) has been used to increase the resistance to viruses such as GFLV, GVA, or GVB (Gölles et al., 1997; Tsvetkov et al., 2000; Gambino et al., 2005). Finally, the introduction of resistance to insects like root-knot nematodes or the grapevine phylloxera have been attempted by means of hairy roots transformation (Franks et al., 2006; Yang et al., 2013). In addition, the improvement of tolerance to abiotic stress has also been studied. Some of the most troublesome stress problems that have been addressed are resistance to cold (Jin et al., 2009; Tillett et al., 2012) or different sources of oxidative damage (Zok et al., 2009).

**TABLE 1:** Genetic transformation works focused on the incorporation of genes related to fungal, bacterial, viral resistance, abiotic stresses, and other pathogens in *V. vinifera*.

Goal	Integrated sequence	Cultivar	Type of explant	Transformation method	*Agrobacterium* strain	Reporter gene	Antibiotics	References
Resistance to grapevine fanleaf virus (GFLV)	CP (chimeric Coat Protein gene)	Chardonnay	Embryogenic cell suspensions (from anthers)	*Agrobacterium* infection	LBA4404	β-glucuronidase (GUS)	Kanamycin	Mauro et al., 1995
Resistance to viruses and bacteria	TomRSV-CP (Tomato RingSpot Virus Coat Protein)/Shiva-1 (lytic peptide gene)	Thompson Seedless	Somatic embryos (from leaves)	Biolistic transformation and *Agrobacterium* infection	EHA101/EHA105	β-glucuronidase (GUS)	Kanamycin	Scorza et al., 1996
Resistance to GFLV and Arabis Mosaic Virus	GFLV CP (Grapevine FanLeaf Virus Coat Protein)/ArMV CP (Arabis mosaic virus Coat Protein)	Rusalka	Embryogenic callus (from immature ovules and vegetative tissues of anthers)	*Agrobacterium* infection	LBA4404	β-glucuronidase (GUS)	Kanamycin	Gölles et al., 1997
Resistance to fungi	Glucanase and chitinase/chitinase and RlP (Ribosome Inactivating Protein)	Riesling, Dornfelder and Müller-Thurgau	Somatic embryos (from anther)	*Agrobacterium* infection	LBA4404	β-glucuronidase (GUS)	Kanamycin	Harst et al., 2000
Resistance to GFLV	GFLV CP (Grapevine FanLeaf Virus Coat Protein)	Rusalka	Embryogenic cultures (from immature zygotic embryos and leaves)	*Agrobacterium* infection	LBA 4404/GV3101	β-glucuronidase (GUS)	Kanamycin	Tsvetkov et al., 2000
Resistance to powdery mildew and anthracnose	*RCC2* (Rice Chitinase gene)	Neo Muscat	Embryogenic callus (from ovules)	*Agrobacterium* infection	LBA4404	–	Kanamycin	Yamamoto et al., 2000
Resistance to fungi	*SP* (Signal Peptide from pea vicilin protein)/*mag2* (magainin class gene)/PGL (Peptidyl-Glycine- Leucine)	Chardonnay	Embryogenic cell suspensions (from anthers or ovaries)	Biolistic transformation	–	β-glucuronidase (GUS)	Kanamycin	Vidal et al., 2003
Resistance to bacterial diseases	*mag2* (natural magainin-2)/*MS199* (a synthetic derivate)	Chardonnay	Embryogenic cell suspensions	Biolistic transformation	–	β- glucuronidase (GUS)	Kanamycin	Vidal et al., 2006
Resistance to *Botrytis cinerea*	*pPgip* (pear Polygalacturonase-inhibiting protein gene)	Chardonnay and Thompson Seedless	Embryogenic callus (from anthers)	*Agrobacterium* infection	EHA 101	β-glucuronidase (GUS), pear polygalacturonase inhibiting protein gene (PGIP), green fluorescent protein gene (*GFP*)	Kanamycin	Agüero et al., 2006
Resistance to *Uncinula necator* and *Plasmopara viticola*	Chitinase and RIP (Ribosome-Inactivating Protein from *Hordeum vulgare*)	Seyval blanc	Leaf disks	*Agrobacterium* infection	LBA 4404	–	Kanamycin	Bornhoff et al., 2005
Resistance to GFLV	GFLV CP (Grapevine FanLeaf Virus Coat Protein)	Nebbiolo Lumassina and Blaufränkisch	Embryogenic callus (from anthers and ovaries)	*Agrobacterium* infection	LBA4404	–	Kanamycin	Gambino et al., 2005
Resistance to phylloxera	*CYP79A* and *CYP71E1* (cytochrome p450 from Shorgum)/s*bHMNGT* (UDPG glucosyltransferase- from Shorgum)	Sultana	Embryogenic callus and whole plants to generate hairy roots	*Agrobacterium* infection	EHA105/A4	Green fluorescent protein gene (*GFP*)	Kanamycin	Franks et al., 2006
Resistance to fungal diseases	*STS* (stilbene synthase gene)	Thompson Seedless	Embryogenic callus (from anthers)	*Agrobacterium* infection	GV3101	β- glucuronidase (GUS), green fluorescent protein gene (*GFP*)	Hygromycin	Fan et al., 2008
Resistance to cold stress	*AtDREB1b* (dehydration response element binding transcription factor in *Arabidopsis thaliana*)	Centennial Seedless	Leaf disks	*Agrobacterium* infection	LBA4404	–	Kanamycin	Jin et al., 2009
Tolerance to abiotic stress	Ferritin gene (*MsFer*) from *Medicago sativa* (alfalfa)	Transgenic *Vitis* berlandieri × *Vitis* rupestris cv. ‘Richter 110’ grapevine rootstock lines	Embryogenic callus (from anthers)	*Agrobacterium* infection	EHA 105	–	Kanamycin	Zok et al., 2009
Tolerance to powdery mildew	*Chi11* (rice chitinase gene)	Pusa Seedless	Embryogenic callus (from leaves)	*Agrobacterium* infection	LBA4404	–	Hygromycin	Nirala et al., 2010
Resistance to powdery mildew, black rot, and sour-bunch rot	*vvtl-1* (*Vitis vinifera* thaumatin-like protein)	Thompson Seedless	Somatic embryos (from leaves)	*Agrobacterium* infection	EHA 105	Green fluorescent protein gene (*GFP*)	Kanamycin	Dhekney et al., 2011
Resistance to Pierce’s disease	PGIP (signal peptide with a lytic domain derived from cecropin)	Thompson Seedless	Embryogenic callus	*Agrobacterium* infection	EHA 105	β- glucuronidase (GUS)	Kanamycin	Dandekar et al., 2012
Tolerance to *Plasmopara viticola*	Chitinase and β-1,3-glucanase	Crimson Seedless	Somatic embryos (from leaves)	*Agrobacterium* infection	LBA4404	–	Kanamycin	Nookaraju and Agrawal, 2012
Resistance to water stress	*VvPIP2;4N* gene (PIP-type aquoporine gene)	Brachetto	Embryogenic callus	*Agrobacterium* infection	LBA4404	–	Kanamycin	Perrone et al., 2012
Tolerance to freezing	*VvCBF4* (C-repeat binding factor gene)	Freedom	Embryogenic callus (from immature anthers)	*Agrobacterium* infection	EHA 105	–	Hygromycin	Tillett et al., 2012
Resistance to Root-Knot nematodes	*pART27-42* (RNA interference silencing a conserved Root-Knot nematode effector gene *16D10/pART27-271*)	Chardonnay	Hairy roots	*Agrobacterium* infection	A4	–	Kanamycin	Yang et al., 2013
Resistance to *Botrytis cinerea*	VstI (grapevine stilbene synthase)	Sugraone	Embryogenic callus	*Agrobacterium* infection	EHA105	Green fluorescent protein gene (*GFP*)	Kanamycin	Dabauza et al., 2014
Resistance to *Botrytis cinerea* and *Erysiphe necator*	*ech42* (endochitinase)/*ech33* (endochitinase)/*nag70* (*N*-acetyl-*b*-Dhexosaminidase gene)	Thompson Seedless	Somatic embryos (from leaves)	*Agrobacterium* infection	EHA105	–	Kanamycin	Rubio et al., 2015
Resistance to Pierce’s disease	LIMA-A (synthetic gene encoding a lytic peptide)	Thompson Seedless	Somatic embryos (from leaves)	*Agrobacterium* infection	EHA105	Green fluorescent protein gene (*GFP*)	Kanamycin	Li et al., 2015
Resistance to powdery mildew	VpSTS (*Vitis pseudoreticulata* stylbene synthase)	Chardonnay	Embryogenic callus, proembryonic masses, somatic embryos (anthers, ovaries and whole flowers)	*Agrobacterium* infection	GV3101	–	Hygromycin	Dai et al., 2015
Resistance to powdery mildew	VpPR4-1 (pathogenesis-related protein from *Vitis pseudoreticulata*)	Red Globe	Pro-embryonic masses (from immature stamens)	*Agrobacterium* infection	GV3101	–	–	Dai et al., 2016
Resistance to downy mildew disease	VaTLP (thaumatin-like protein related to pathogenesis)	Thompson Seedless	Pre-embryogenic callus (anthers)	*Agrobacterium* infection	EHA105	–	Kanamycin	He et al., 2016
Resistance to powdery mildew	*VpRH2* (RING-H2 type ubiquitin ligase gene)	Thompson Seedless	Somatic embryos	*Agrobacterium* infection	GV3101	β-glucuronidase (GUS)	–	Wang et al., 2017
Tolerance to *Plasmopara viticola*	*VpPR10.1* (pathogenesis-related gene)	Thompson Seedless	Pro-embryonic masses (from anthers)	*Agrobacterium* infection	GV3101	Green fluorescent protein gene (*GFP*)	Kanamycin	Su et al., 2018

A compilation of most of the works carried out on stable transformation of *V. vinifera*, including the transformation methods, marker genes, and antibiotics used for selection are shown in Table 1. It is worth mentioning that most of the grapevine genetic transformation studies have used varieties such as “Thompson Seedless” or “Chardonnay,” highlighting the genotype-specificity of this procedure.

## New Technologies and Strategies to Improve the Transformation and Regeneration in Grapevine

Plants are sessile organisms that are dependent on the living conditions of the environment around them, and for this reason plants have developed great plasticity to accommodate environmental effects by altering metabolism or development (Fehér et al., 2003). Pluripotency and totipotency, exceptional properties for tissue culture techniques, have contributed to several biotechnological applications. Pluripotency refers to the ability of one cell type to form another cell type, tissue, or organ, while totipotency refers to the ability of a single cell to develop, through embryogenesis, into a complete organism (Jha and Kumar, 2018). The totipotency theory was first proposed by Guttenberg (1943), but regeneration protocols were established after Skoog and Miller (1957) introduced changes in the concentration of auxins and cytokinin in the culture media. Since then, it has been possible to establish shoot regeneration for many plants (Lardon and Geelen, 2020).

The most common types of regeneration in plants are somatic embryogenesis and *de novo* organogenesis (Pulianmackal et al., 2014; Kareem et al., 2016). During somatic embryogenesis, dedifferentiated cells generate bipolar structures where it is possible to differentiate root and shoot meristems (Pulianmackal et al., 2014; Xu and Huang, 2014; Horstman et al., 2017; Méndez-Hernández et al., 2019). This process is achieved through abiotic stress induction or by the addition of auxins. Consequently, zygotic embryogenesis-like structures are formed, due to the action of transcription factors such as LEAFY COTYLEDON (LEC) 1 and 2, AGAMOUS- LIKE 15 (AGL15), FUSCA 3 (FUS3), BABYBOOM (BBM), and EMBRYOMAKER (EMK) (Horstman et al., 2017; Méndez-Hernández et al., 2019). On the other hand, *de novo* organogenesis consists of the formation of new meristems from pluripotent stem cells to build organs (Xu and Huang, 2014). This process is governed by the plant hormones auxin and cytokinin and a transcriptional cascade involving WUSCHEL-RELATED HOMEOBOX (WOX) 11 and 12, WOX5 and 7, and LATERAL ORGAN BOUNDARY DOMAIN (LBD) 16 and 29, and SHOOT MERISTEMLESS (STM) (Liu et al., 2014; Xu, 2018).

On the other hand, GROWTH-REGULATING FACTOR (GRF) genes were reported as plant-specific transcription factors involved in the establishment and maintenance of meristems and in the cellular proliferation of developing primary organs (Liebsch and Palatnik, 2020). GRF proteins interact with a transcription cofactor, GRF INTERACTION FACTOR (GIF), forming a functional transcriptional complex (Kim and Kende, 2004). GIFs can act as transcriptional coregulators enhancing the activity of GRFs. MicroRNA396 (miR396) is a conserved miRNA that recognizes a complementary sequence in GRF mRNA from seed plants. Finally, GRF expression is regulated by posttranscriptional repression by mir396 (Rodriguez et al., 2016). The combinatory action of the miR396-GRF/GIF system in regulating plant growth makes it a very valuable tool for improving crops of agronomic interest.

### Role of Transcription Factors in Plant Transformation and Regeneration

Grapevine, as many plant species present difficulties in transformation and regeneration. These varieties are said to be recalcitrant to being transformed and regenerated. One of the promising tools that helps reduce these difficulties is the use of genes involved in the control of plant growth and development, called developmental regulators or morphogenetic regulators. Increases in the efficiency of the transformation and regeneration of various plants using developmental regulators have been thoroughly reported (Srinivasan et al., 2007; Heidmann et al., 2011; Yang et al., 2014; Lutz et al., 2015; Lowe et al., 2016; Mookkan et al., 2017; Maher et al., 2020; Che et al., 2021).

The overexpression of the *WUSCHEL* (*WUS*) gene has been used in several models and species of crops with the aim of improving the efficiency of transformation (Lowe et al., 2016; Mookkan et al., 2017; Che et al., 2021). Lowe et al. (2016) reported in maize, a significant increase in the frequency of callus transformation with *WUS2*. Moreover, the combination of *WUS2* and *BBM* led to the highest transformation frequency. Mookkan et al. (2017) reported that the coexpression of the *BABY BOOM* (*BBM*) and *WUS2* maize transcription factors along with a desiccation inducible CRE/lox cleavage system allows the regeneration of inbred stable recalcitrant transgenic maize B73 and sorghum P898012 without a selectable chemical marker. An increase in the transformation frequency from 0 to 15% for the B73 genotypes and upto 6.2% for the P898012 genotypes was found without the use of selection agents. This selectable-marker-independent transformation may contribute to overcoming transformation barriers in recalcitrant species and facilitate studies using gene editing functions. More recently, Che et al. (2021) reported that the transformation of *Wus2* is capable of increasing the efficiency in the regeneration of transgenic plants and the efficiency in genome editing through the CRISPR-Cas technology. In addition, the authors have developed advanced cleavage systems and transformation technology to generate high quality selectable-marker-free sorghum events and/or morphogenic genes. They conclude that *Wus2*-enabled genome editing may be applicable to other crops in plant transformation strategies. On the other hand, *BBM* was reported as a marker and an activator of a complex signaling network of different development pathways related to cell proliferation and growth (Passarinho et al., 2008). Overexpression of native and heterologous *BBM* genes has also been found to play a role in inducing cell proliferation and significantly improving transformation and regeneration efficiency in tobacco (Srinivasan et al., 2007), oil palm (Morcillo et al., 2007), Arabidopsis (Lutz et al., 2015), and dog rose (Yang et al., 2014).

Alternatively, to improve plant regeneration rates after gene transformation, Debernardi et al. (2020) demonstrated that the expression of a fusion protein that combines the wheat GROWTH REGULATORY FACTOR 4 (GRF4) and its cofactor INTERACTIVE FACTOR GRF 1 (GIF1) was capable of increasing regeneration when it is expressed in crops such as wheat, triticale, and rice (Table 2). The authors also evaluated the use of the GRF4–GIF1 system together with the CRISPR-Cas9 gene editing technology by designing a cassette that included the GRF4-GIF1 chimera, Cas9 and, a guide RNA (gRNA) directed to the wheat Q gene (also known as AP2L-A5). Debernardi et al. (2020) were able to recover transgenic events including seven fertile plants showing a higher number of florets per spikelet (characteristic of q-null plants). The efficiency of the GRF–GIF chimera was also tested in citrus transformation experiments by means of the generation of a citrus GRF-GIF chimera and a heterologous GRF–GIF grapevine chimera (Debernardi et al., 2020). The epicotyls transformed with both chimeras showed significant increase in the frequency of regeneration compared with those transformed with the empty vector control (Table 2).

**TABLE 2:** Comparative summary of the different transcription factors used in different crops and model plants (Debernardi et al., 2020; Maher et al., 2020).

Crop		Transcription factor combinations	Best transcription factor combinations	Reporter gene	Transformed plant material	Edited gene	Observed phenotype	Time consumed (days)	Average regeneration
*Nicotiana benthamiana* harboring a 35S:Cas9 transgene		All combo = *Wus2*/STM/*BBM*/ *MP*Δ/*ipt*, *Wus2* + *ipt*, *Wus2* + *STM*, *ipt*	*Wus2* + *ipt*, *ipt* alone, All combo	Luciferase	Soil-grown plants	–	Distorted morphology and luminescence	62	Data not shown
*Nicotiana benthamiana* harboring a 35S:Cas9 transgene		*Wus2*/*ipt, Wus2* + *ipt*	*Wus2/ipt*	Luciferase	Soil-grown plants	*PDS*	Green, green and white chimeric, white and distorted shoots	Data not shown	Data not shown
*Vitis vinifera* (Pixie Pinot Meunier Purple)		*nos*:Zm*Wus2* + *35S:ipt* + *35S:MP* Δ + *35S:STM* + *AtUbi10:BBM*	*nos*:Zm*Wus2* + *35S:ipt* + *35S:MP*Δ + *35S:STM* + *AtUbi10:BBM* (Unique combination tested)	Luciferase	Soil-grown plants	–	Normal transgenic shoots	40	Data not shown
*Solanum tuberosum* (Ranger Russet)		*ipt, ipt/Wus2*	Data not shown	Luciferase	Soil-grown plants	–	Abnormal shoots and transgenic shoots	100	Data not shown
Wheat	Kronos	*GRF4-GIF1, GRF4–GIF2, GRF4–GIF3, GRF5–GIF1, GRF1–GIF1, GRF9–GIF1*	*GRF4-GIF1*	–	Immature embryos	–	Transgenic normal and fertile wheat plants	60	65.1
	Desert King	*GRF4-GIF1*	*GRF4-GIF1* (Unique combination tested)	–	Immature embryos	–	Green shoots	60	63
	Fielder	*GRF4-GIF1*	*GRF4-GIF1* (Unique combination tested)	–	Immature embryos	–	Green shoots	60	62
	Cadenza	*GRF4-GIF1*	*GRF4-GIF1* (Unique combination tested)	–	Immature embryos	–	Normal and fertile wheat plants	60	19
	Hahn	*GRF4-GIF1*	*GRF4-GIF1* (Unique combination tested)	–	Immature embryos	–	Green shoots	60	9
	Kronos	*GRF4–GIF1/CRISPR–Cas9–gRNA–Q*	*GRF4–GIF1/CRISPR–Cas9–gRNA–Q* (Unique combination tested)	–	Immature embryos	gene *Q* (*AP2L-A5*)	Plants with an increased number of florets per spikelet	60	93,7
Triticale	Breeding line UC3184	*GRF4-GIF1*	*GRF4-GIF1* (Unique combination tested)	–	Immature embryos	–	Green shoots	60	10
Citrus		*Citrus GRF-GIF*	*Vitis rGRF4-GIF1*	–	*Citrus* epicotyls	–	Mostly normal shoots	60	21
		*Vitis GRF-GIF*		–	*Citrus* epicotyls	–	Mostly normal shoots	120	16
		*Vitis rGRF4-GIF1*		–	*Citrus* epicotyls	–	Normal and abnormal shoots	120	37
Rice Kitaake		*GRF4-GIF1*	*GRF4-GIF1* (Unique combination tested)	–	Callus	–	Shoots	70–80	43

Finally, taking advantage of totipotency and pluripotency of plants, the ectopic expression of specific transcription factors called development regulators (DR) has the potential to induce meristems in somatic cells. Maher et al. (2020) presented the successful genome editing by *de novo* reprogramming of plant meristems in somatic tissues, which avoids tissue culture-based transformation and promises to significantly improve the utility of gene editing in plants. This innovative work proposes the induction of *de novo* meristems on soil-grown plants. *N. benthamiana* plants that constitutively expressed Cas9 were cultivated until the apical and axillary meristems were clearly differentiated, the point when they will be removed. DR combinations were delivered by *A. tumefaciens* at the breakpoints. Over time, *de novo* gene-edited shoots were formed, and editing events are passed to the next generation. Maher et al. (2020) also test the system in grapevine potato plants (Table 2).

### Carbon-Based Systems as a New Technology for Biomolecules Delivery

To date, plant biotechnology lacks a method that allows passive delivery of diverse biomolecules without the aid of external force. As discussed previously, traditional methods present host-range limitations and typically target immature plant tissue (calli, meristems, or embryos), while efficient protocols have only been developed for a narrow range of plant species.

In this era, nanotechnology applications in agriculture have quickly emerged since they have little impact on environment. Due to the large surface area, tunable pore size, cargo and structure, and their tailored functionality, nanomaterials are widely used as nanoparticle-based fertilizers (Liu and Lal, 2015); antimicrobial components like silver and copper nanoparticles (Morones et al., 2005; Borkow and Gabbay, 2009; Sharon et al., 2010; Adeleye et al., 2016; Keller et al., 2017); and nanotechnology, which is being extensively applied in the genetic modification of plant DNA (Ziemienowicz et al., 2012). Nanotechnology has become a promising genetic cargo delivery toolset that is (i) plant-species independent (Su et al., 2019); and (ii) capable of high performance despite the physical barriers presented in intact plant tissues such as the plant cell wall (Etxeberria et al., 2006; Liu et al., 2009; Demirer et al., 2019a). The use of nanotechnology in gene modification enables easy operation, high efficiency (1,000 times less DNA is needed compared to conventional DNA modification techniques), versatility (nanoparticles are capable of simultaneously introducing proteins, nucleotides, and chemicals), target-specific delivery, and on-site release (Climent et al., 2009; Lin et al., 2015; Milewska-Hendel et al., 2017).

Compared with other metal-based nanomaterials, carbon-based nanomaterials show much lower environmental toxicity and higher biocompatibility due to their non-toxic carbon backbone (Chen et al., 2015; Bhattacharya et al., 2016; Mukherjee et al., 2016). Additionally, they have variety of sizes and shapes (including nanosheets, nanotubes, nanodots). Herein, carbon-based nanomaterials have become very versatile and sustainable materials, thus they have been widely applied in agriculture (Mukherjee et al., 2016; Shojaei et al., 2019; Verma et al., 2019). CDs mainly include graphene quantum dots (GQDs), carbon nanodots (CNDs), and polymer dots (PDs). These nanoparticles possess a size of less than 10 nm and have inherent photoluminescence (PL) and photostability properties, biocompatibility, abundant source, water solubility, highly tunable PL properties, easy functionalization with biomolecules, and chemical inertness (Li et al., 2012; Zheng et al., 2015; Peng et al., 2017). This kind of carbon material is much smaller than carbon nanosheets and nanotubes, allowing the CDs to pass much more easily through the biofilm of the cells of a broad range of plant phenotypes and species, including immature plant tissue and mature plants (model organisms, crop plants, and orphan crops -plants notoriously recalcitrant to transformation-) (Schwab et al., 2016; Tripathi et al., 2017). Moreover, it avoids the use of antibiotics in culture media, overcoming the loss of viability of plant tissues and accelerating the regeneration processes and has no effect on photosynthesis or growth of transformed crops and provokes no damage (Doyle et al., 2019; Li et al., 2020). Therefore, we present CD as a promising alternative that can still be used to improve the transformation and regeneration of the grapevine. Although not much work of this type has been carried out in grapevine, we consider this technique suitable to cope with more sustainable transformation protocols, which would lay the foundations for genetic improvement of grapevine in the era of food and nutrition security.

Carbon-based system for the delivery of cargo (RNA, DNA, protein, and plant protection substances) into the plant cell, is gaining relevance as it is easy, fast, and inexpensive to manufacture, requires little equipment to make, and can be adapted to a variety of application strategies to obtain genetically modified plants (Table 3). For instance, CD–plasmid nanocomplexes can act as a delivery vehicle by which plasmids can be carried into plant somatic cells, allowing transient expression. In the work of Doyle et al. (2019), plasmid-coated PEG functionalized CDs were sprayed on wheat, maize, barley, and sorghum leaves. CD-plasmid complexes containing *GFP* gene with a nuclear localization sequence (NLS) were successfully introduced and transiently expressed into the nucleus. The plasmid also carried the Cas9 gene and gRNA to make a ∼250 bp deletion in the wheat *SPO11* genes. Importantly, spraying CD-plasmid nanocomplexes onto intact leaves can edit the genome. Similarly to Wang et al. (2020), PEI-modified CDs (CDP) with a positive charge, provides a highly efficient CD-based DNA delivery system for rapid and transient gene expression. Hygromycin resistance was achieved smearing plants leaves or soaking roots of rice with CD-plasmid complexes containing hydamycin resistance gene, whereas dipping and vacuum mature rice embryo with CD-plasmid complexes containing β-glucuronidase induced callus.

**TABLE 3:** Carbon-based nanoparticles NPs as biomolecule carriers for transient expression.

Carbon-based NPs	Plant species	Modes of application	Genetic modification	References
PEG functionalized CDs	Wheat, maize, barley, and sorghum	Spray on leaves	– Transient expression of GFP, Cas9, gRNA -Edition of SPO11 genes through Cas9	Doyle et al., 2019
PEI-modified CDs (CDP)	Rice	– Smearing plants leaves and soaking roots of mature rice plants -Dipping and vacuum mature rice embryo induced callus	– Transient expression of Hydamycin resistance gene and β-glucuronidase	Wang et al., 2020
SWCNTs	*Nicotiana tabacum*	Incubation of protoplasts with NPs solution	– Transient expression of *yfp* reporter gene	Burlaka et al., 2015
MWCNTs	*Nicotiana tabacum*	Incubation of protoplasts and leaf explants treated by carborundum with NPs solution	– Transient expression of *nptII* gene	Burlaka et al., 2015
CNTs	Arugula, watercress, spinach, tobacco and *Arabidopsis thaliana*	Incubation of mesophyll protoplasts and infiltration of leaves	– Transient expression of *yfp* reporter gene	Demirer et al., 2019b
SWCNTs MWCNTs	*Nicotiana benthamiana*, arugula, wheat, and cotton	Infiltration of leaves and incubation of protoplasts	– Transient expression of GFP-encoding DNA plasmids or linear PCR amplicons	Kwak et al., 2019
PEI-modified CDs (CDP)	*Nicotiana benthamiana* and tomato	Low-pressure spray + spreading surfactant leaves	– siRNA for silencing GFP transgene -siRNA for silencing two subunits of endogenous magnesium chelatase	Schwartz et al., 2020

Nanosized carriers, like the oxidized multiwalled carbon nanotubes (MWCNTs) and single-walled carbon nanotubes (SWCNTs), were also studied to deliver DNA into mesophyll protoplasts, callus cells, and leaf explants. Thus, *N. tabacum* protoplasts were genetically transformed with the plasmid construct pGreen 0029, and a transient expression of the *YFP* reporter gene was shown in the protoplasts (Burlaka et al., 2015). While *N. tabacum* callus and leaf explants were genetically transformed by the *nptII* gene contained in the pGreen 0029 construct, regenerated plants were obtained on a selective medium. The investigation of Burlaka et al. (2015) demonstrated SWCNTs applicability for the transformation of protoplasts and walled plant cells. At the same time, MWCNTs demonstrated their applicability only for the transformation of protoplasts because of a limiting role of the cellulose wall against their penetration into the cells. Similarly, efficient *GFP*-encoding DNA plasmids or linear PCR amplicons and strong protein expression, without transgene integration, was achieved in *N. benthamiana*, arugula, wheat and cotton leaves, and arugula protoplasts. Demirer et al. (2019a) found that CNTs not only facilitate biomolecule transport into plant cells but also protect polynucleotides from nuclease degradation, without transgene integration. CNTs also have served as carrier of pDNA encoding yfp reporter gene for transient expression in the chloroplasts of mature arugula, watercress, tobacco and spinach plants and in isolated *A. thaliana* mesophyll protoplasts (Kwak et al., 2019).

The delivery of CD-based small interfering RNAs (siRNAs; double-stranded RNAs of 20–25 bp) into plant cells expands the spectrum of carbon-based NPs for molecule delivery into plant cells. RNA-induced gene silencing (also known as RNA interference) is a reliable method to study and alter the genetic form and function of plants. In Schwartz et al. (2020), PEI modified CDs (CDP) were used for delivering siRNA into the model plant *N. benthamiana* and tomato. Low-pressure spray application of these formulations with a spreading surfactant resulted in strong silencing of the reporter gene *GFP* transgenes in both species. The delivery efficacy of CD formulations was also demonstrated by the silencing of endogenous genes that encode two subunits of magnesium chelatase, an enzyme necessary for chlorophyll synthesis.

A breakthrough has been achieved in transient expression in plant somatic and embryogenic cells and protoplasts using carbon-based NPs as delivery method. This approach is very promising as it could be used to express genes and silence or increase gene expression, without gene integration, which would be particularly useful for plant developmental research. Moreover, it gives hope to the limitations of host restrictions. This kick off leads to more crops being assayed every day to test this new form of genetic cargo delivery. Particularly, for grapevine, only basic research has been carried out using NPs. However, it already stands out with interesting and eligible properties. For instance, in the work of Valletta et al. (2014), poly(lactic-*co*-glycolic) acid (PLGA) based CDs were demonstrated to cross the plant cell wall and membrane of *V. vinifera* cell cultures and grapevine-pathogenic fungi. By means of fluorescence microscopy, PLGA CDs can enter into grapevine leaf tissues through stomata openings so that they can be absorbed by the roots and transported to the shoot through vascular tissues. Viability tests demonstrated that PLGA CDs were not cytotoxic for *V. vinifera*-cultured cells. The cellular uptake of PLGA NPs by some important grapevine-pathogenic fungi shows promising potential for future use in agricultural applications, offering the possibility to deliver chemicals to specific targets in a controlled manner.

In the future, refinement and optimization experiments will surely lead to stable edited gene lines targeting plant germ line cells. Nevertheless, for all types of genetic modification, a comparable high efficacy with less established plant species needs to be shown.

## Conclusion

**FIGURE 1:**
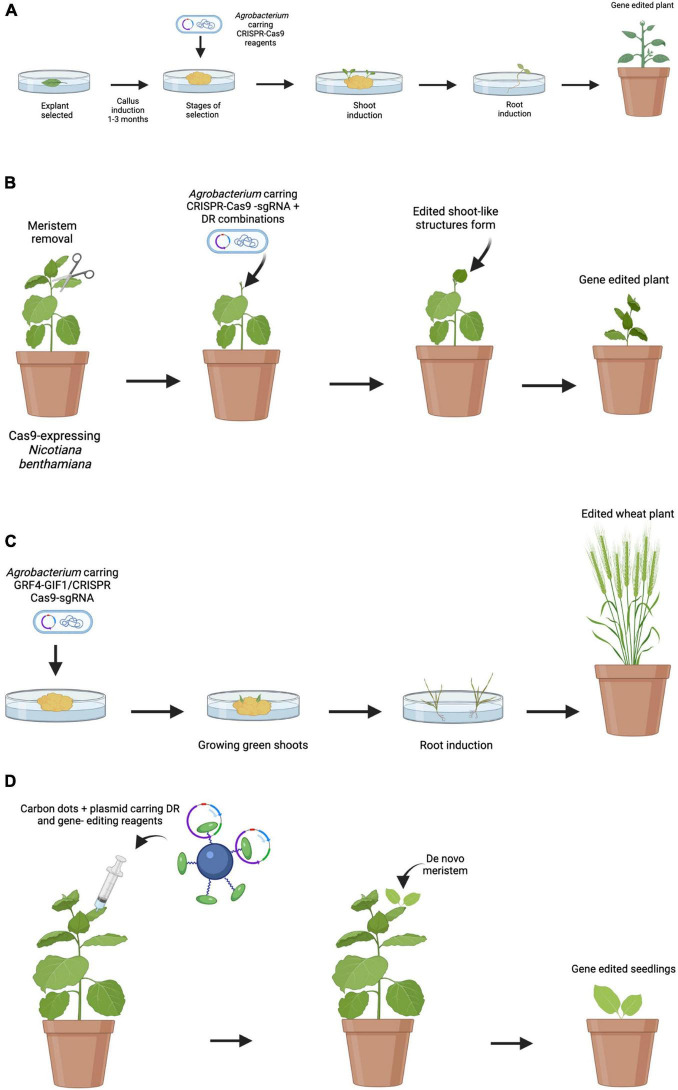
Comparison of traditional and emerging transformation and edition techniques. **(A)** Traditional *Agrobacterium*-mediated embryo transformation. Embryos are obtained and then incubated with *A. tumefaciens*. Multiple steps of selection are done to identify transgenic callus. Selected calli are transferred to shoot induction media follow by root induction media. Finally, plants are transferred to soil. **(B)** Induction of transgenic shoots on soil-grown plants. Meristems are removed, and DRs and gene-editing reagents are delivered by *A. tumefaciens*. After a while, *de novo* gene-edited shoots are formed and editing events are transmitted to the next generation. **(C)** Induction of edited shoots using the GRF–GIF chimera. GRF4–GIF1/CRISPR–Cas9–gRNA construction is delivered by *A. tumefaciens*. As a result, an increase in regeneration efficiency is observed. The shoots are then transferred to a medium to root and develop into whole plants. **(D)** Proposed model for nanoparticle mediated CRISPR/Cas9 in plant engineering. Nanoparticles can deliver DR and CRISPR/Cas9 reagents into plant cells, resulting in transgenic plants through *de novo* induction of meristems.

Up to now, most of the work carried out in grapevine genetic transformation has had relative success due to the problems related to the transformation processes and the difficulty in plant regeneration. This process is generally time-consuming and involves several laborious steps (Figure 1A).

As shown in the present review, the preferred transformation method for stable grapevine transformation is *Agrobacterium*, and that most of the works have focused mainly on the use of few grapevine cultivars like “Chardonnay” and “Thompson Seedless.” Regarding the use of antibiotics for the selection of transformants, the determination of the optimal concentration for each genotype emerged as a necessary step since antibiotic sensitivity showed to be genotype-dependent. Accordingly, the possibility of using reporter genes such as *GFP* or *RUBY* as a control of the transformation appears as an attractive alternative. Therefore, the use of new technologies and their combination is required to facilitate the recovery of many plants in a large number of grapevine cultivars. In this sense, the application of the technologies proposed by Debernardi et al. (2020) and Maher et al. (2020) would be very useful to increase regeneration rates (Figures 1B,C). As previously mentioned, the tissues used for transformation are sensitive to infection with *A. tumefaciens*. For this reason, the use of nanoparticles-derived delivery systems, such as CDs, emerges as an alternative to overcome this problem with the advantage that it would allow to extend the range of hosts. This work proposes the possibility of combining the technologies developed by Debernardi et al. (2020) and Maher et al. (2020) together with the delivery of vectors mediated by nanoparticles, with the aim of overcoming problems and limitations related to the classical methodology of grapevine transformation and plant regeneration (Figure 1D).

## Author Contributions

GC and CC wrote the manuscript. SM provided critical feedback. DL conceived and performed the final edition of the manuscript. All the authors approved the final version of the manuscript.

## Conflict of Interest

The authors declare that the research was conducted in the absence of any commercial or financial relationships that could be construed as a potential conflict of interest.

## Publisher’s Note

All claims expressed in this article are solely those of the authors and do not necessarily represent those of their affiliated organizations, or those of the publisher, the editors and the reviewers. Any product that may be evaluated in this article, or claim that may be made by its manufacturer, is not guaranteed or endorsed by the publisher.
